# Study of the Orbital Circular Cutting in Quartz Wafers Using Electrochemical Discharge Machining with Micro-Electrodes

**DOI:** 10.3390/mi17070832

**Published:** 2026-07-12

**Authors:** A-Cheng Wang, Jung-Chou Hung, Yu-Lun Tsai, Hai-Ping Tsui

**Affiliations:** 1 Department of Mechanical Engineering, Chung Yuan Christian University, Taoyuan City 320314, Taiwan; acwang165@gmail.com; 2 Department of Mechanical Engineering, National Central University, Taoyuan City 320317, Taiwan; hungjc@ncu.edu.tw (J.-C.H.); eddie333445@gmail.com (Y.-L.T.)

**Keywords:** electrochemical discharge cutting machining, quartz wafer, micro-helical electrode, orbital circular cutting, circular path cutting

## Abstract

Quartz wafer dicing technologies primarily rely on mechanical cutting and etching processes. Mechanical cutting is easy to generate the micro-cracks along the wafer edges, which compromises component precision. Furthermore, etching processes are associated with long processing times, high manufacturing costs, and environmental concerns. To address these limitations, this study proposes an electrochemical discharge cutting machining (ECDCM) method using a micro-tungsten carbide helical electrode performing orbital circular cutting (OCC) to evaluate the feasibility and optimization of quartz wafer dicing. Experimental studies were conducted to evaluate the effects of applied voltage, pulse duration, Z-axis feed rate, and duty factor on slot width, slot depth, slot surface quality and tool electrode wear. The results demonstrate that employing an OCC of micro-electrode facilitates the efficient flow of electrolyte into the machining zone, thereby enhancing discharge stability and slot quality. Compared to circular path cutting (CPC) with a rotating electrode, the proposed method reduces machining time by nearly four times and decreases material loss during circular quartz wafer cutting by approximately 50%. These findings indicate that the proposed machining approach provides high efficiency and high-quality quartz wafer cutting.

## 1. Introduction

Quartz wafers possess excellent physical properties, including high ultraviolet (UV) transmittance at specific wavelengths, outstanding piezoelectric characteristics, a low coefficient of thermal expansion, high mechanical strength, unique optical properties, and excellent chemical stability. Owing to these advantages, quartz wafers are widely utilized in applications such as mobile communications, timekeeping devices, artificial intelligence (AI) computing, and satellite navigation systems [1,2]. However, the high hardness and brittle nature of quartz make it difficult to machine. Conventional cutting processes are limited by the width of the cutting tool edge, resulting in relatively large slot widths and consequently significant material loss. Laser cutting is widely used for quartz wafer dicing because it provides a convenient and high-speed method for separating quartz wafers into individual pieces. However, the high thermal energy generated during laser processing may induce a heat-affected zone, micro-cracks, edge chipping, and recast layers, thereby degrading the machining quality and dimensional accuracy of the diced quartz wafers [3]. Although etching processes can also be employed for quartz wafer dicing, this method generally requires considerable processing time and cost and may cause environmental pollution problems [4]. ECDM has recently attracted significant attention as an emerging machining technology due to its superior machining speed and precision, particularly for non-conductive materials [5,6,7,8]. Kim [9] and Zheng et al. [10] proposed replacing direct current (DC) voltage with square wave pulse voltage for micro-hole drilling of Pyrex glass. The results demonstrated that pulse voltage could reduce thermal damage during drilling and improve machining accuracy. In addition, several studies have reported that ultrasonic-assisted ECDM can effectively enhance machining efficiency and accuracy by promoting rapid electrolyte renewal and thinning the insulating gas film through high-frequency ultrasonic vibrations [11,12,13].

Since the gas film generated during the ECDM process strongly influences machining stability, gas film formation has become a critical research topic. Liu et al. [14] employed a rotating helical electrode to improve electrolyte renewal within the machining zone. The results showed that the helical grooves effectively guided the electrolyte toward the machining bottom, enabling the fabrication of micro-slots with high aspect ratios. Similarly, Shi et al. [15] investigated ECDM using a rotating helical electrode and found that increasing the electrode rotational speed resulted in a denser and more uniform gas film, thereby effectively improving the material removal rate (MRR). However, previous studies using helical electrodes have primarily employed a machining strategy consisting of rotational drilling followed by path milling. In this approach, the cylindrical surface of the electrode is responsible for material removal, leading to a relatively large machining area. As a result, the insulating gas film generated during electrochemical discharge machining is difficult to maintain in a stable state, which reduces discharge stability, decreases machining efficiency, and increases the overcut. Wire electrochemical discharge machining (WECDM) utilizes a wire electrode as the cutting tool for electrochemical discharge cutting. Most WECDM processes are performed under submerged conditions, requiring a large amount of electrolyte and involving challenges related to wire breakage [16]. Kong et al. [17] employed a dual-helical wire electrode for WECDM cutting of non-conductive materials. Although the helical wire effectively suppressed gas film diffusion and improved machining stability, the fabrication of helical wire electrodes is difficult and time-consuming. Furthermore, electrode wear is relatively severe, which may adversely affect machining accuracy.

To overcome these limitations, this study proposed a helical electrode that performs orbital motion while advancing downward with a small incremental feed to rapidly follow the prescribed cutting path during quartz wafer dicing. Unlike the conventional rotational milling approach, material removal is primarily accomplished by the drill tip, resulting in a much smaller effective machining area. Consequently, the thickness of the insulating gas film generated during the electrochemical discharge process can be more effectively controlled, leading to improved discharge stability. As a result, both the machining efficiency and cutting accuracy are significantly enhanced.

## 2. Materials and Methods

### 2.1. Experimental Setup

**Figure 1:**
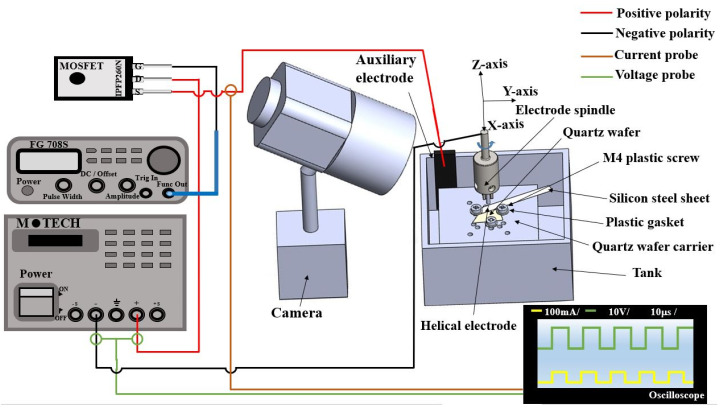
The experimental setup in ECDCM.

**Figure 2:**
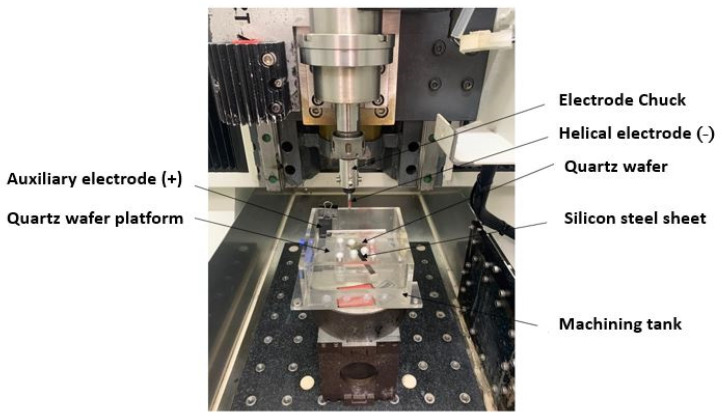
Photograph of the experimental ECDM system.

**Figure 3:**
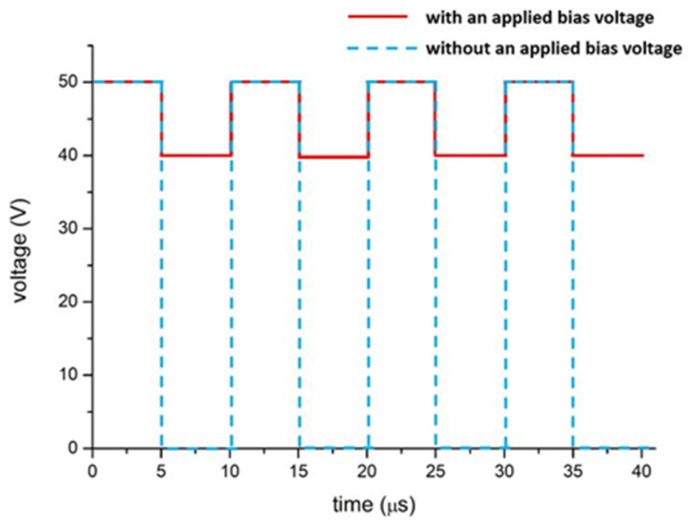
Schematic diagram of pulse composite bias.

The experimental setup is illustrated in Figure 1. The experiments were conducted using an ECDM system (CHMER, E46), which is equipped with a programmable feed control system to accurately regulate the electrode feed rate during machining. The pulse voltage is adjustable from 1 to 70 V, covering the operating voltage range required for both electrochemical machining (ECM) and electrochemical discharge machining (ECDM). A photograph of the experimental ECDM system is presented in Figure 2. In the experiment, a helical drill electrode was mounted onto the machine spindle; this electrode was secured to the spindle by means of an O-ring, with the rotational speed controlled via NC program. The machining power was supplied by a DC power supply (MoTech, PPS-2018A). The positive polarity was connected to the graphite electrode, while the negative polarity was connected to the helical tool electrode. To generate the pulsed bias voltage, a signal generator (MoTech, FG-708S, New Taipei City, Taiwan) and a MOSFET (Infineon, IPFP260-N, Nijmegen, Netherlands) were utilized to modulate the DC output, as depicted in Figure 3. The working current was monitored using an oscilloscope (KEYSIGHT, InfiniVision DSOX3022T, Hsinchu County, Taiwan). A CCD imaging system (SAGE Vision, SG-201L) was employed to observe and record the quartz wafer cutting process in situ. After cutting, the slot morphology and helical electrode shapes were characterized by a white-light interferometer (KEYSIGHT, VK-X3000, Hsinchu County, Taiwan). The same instrument was also utilized to measure the slot dimensions. Furthermore, morphological variations in the slots after quartz wafer cutting were investigated using a scanning electron microscope (JEOL, JSM-7000F, Hsinchu County, Taiwan), which was employed to acquire detailed images of the cutting edge.

### 2.2. Materials

**Figure 4:**
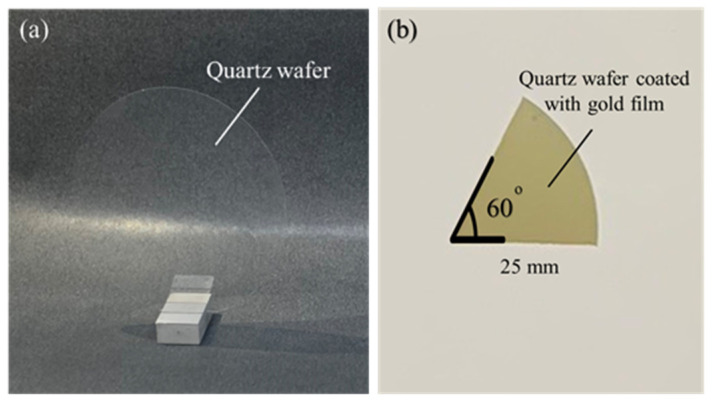
Diagrams of quartz wafer: (**a**) 2-inch circular quartz wafer; (**b**) sector-shaped quartz wafer coated with gold film.

**Figure 5:**
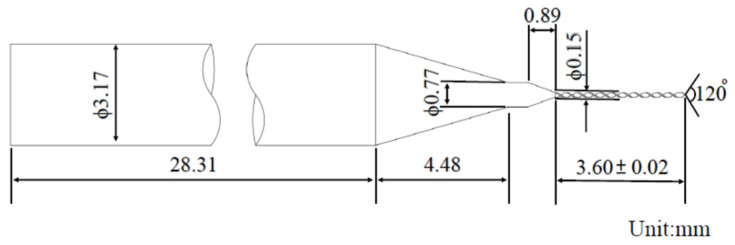
Dimensions of helical electrode.

The workpiece was a single-crystal quartz wafer with a diameter of 2 inches and a thickness of 70 μm, as shown in Figure 4a. The quartz wafer was divided into six sector-shaped pieces before machining, each corresponding to one-sixth of the original wafer area, and a gold coating was deposited on the wafer surface, as shown in Figure 4b. The helical electrode used for machining was a micro-drill with a diameter of 150 μm. The micro-drill was fabricated from tungsten carbide due to its high strength and high melting point. The dimensions of the helical electrode were illustrated in Figure 5. A 5 M potassium hydroxide (KOH) solution was used as the electrolyte, and ethanol was added to the KOH solution at a volume ratio of 9:1 (KOH) to improve the wettability of the helical electrode surface [17,18]. The electrolyte composition was selected based on the findings reported in the above references, while the ethanol ratio adopted in this study was determined through preliminary experiments conducted during the initial stage of the research. In addition, graphite, owing to its excellent chemical stability, was selected as the auxiliary electrode throughout the experiments.

### 2.3. Experimental Procedures

**Figure 6:**
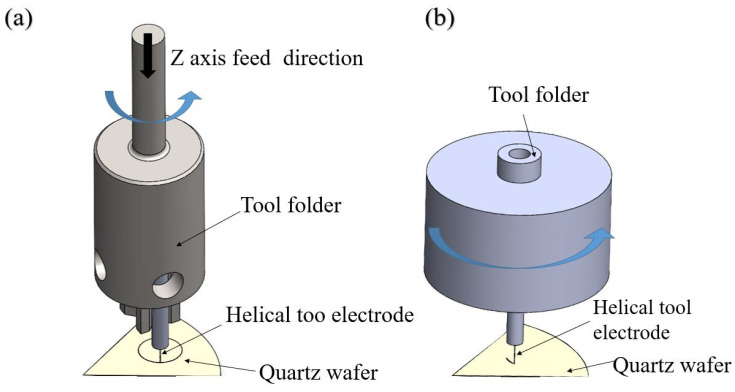
Two machining methods for circular quartz specimens: (**a**) orbital circular cutting (OCC); (**b**) circular path cutting (CPC).

Prior to the experiment, the gold-coated quartz wafer with a sector shape was fixed onto the fixture platform inside the machining tank (Figure 1) by plastic screws and washers. To determine the surface position of the quartz wafer, a 40 μm thick silicon steel sheet was placed beneath the plastic washer of one fixing screw, allowing the wafer height to be determined using a digital multimeter. Two cutting strategies were adopted for fabricating circular quartz specimens. In the first approach, the tool electrode remained stationary without self-rotation, while the spindle performed orbital motion at 50 rpm along a circular trajectory. Simultaneously, the Z-axis was fed downward layer-by-layer at a feed rate of 1/5 μm/s during the OCC process, resulting in the fabrication of a circular quartz piece with a diameter of 8 mm, as illustrated in Figure 6a. In the second approach, the tool electrode was first rotated at 500 rpm, followed by drilling into the quartz wafer with a Z-axis feed rate of 20 μm/s, until to penetrate the workpiece. Subsequently, CPC with a diameter of 8 mm was performed using the same feed rate, as illustrated in Figure 6b. ECDCM parameters used for circular quartz piece cutting are summarized in Table 1.

**Figure 7:**
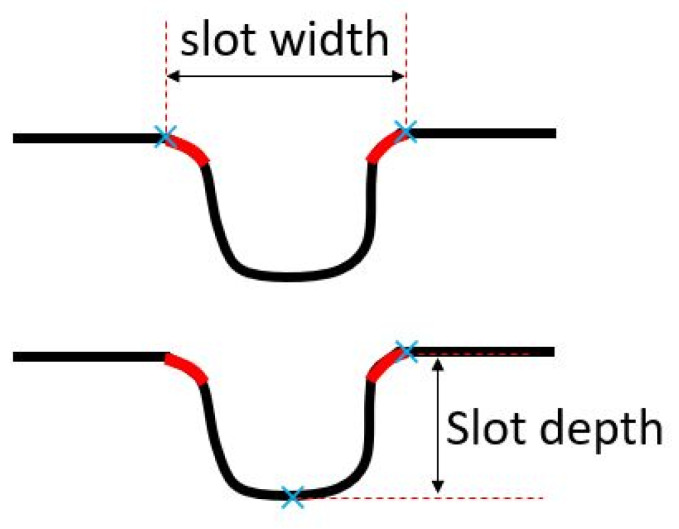
Diagram of measuring slot width and slot depth.

After comparing the OCC process and CPC process, the effects of machining parameters, including working voltage, pulse duration, Z-axis feed rate, and duty factor, on slot depth and slot width during OCC were further investigated. To ensure the reliability of the experimental results, each experimental condition was repeated three times. ECDCM parameters for OCC are listed in Table 2. Since slot width and slot depth directly affect machining accuracy, measurement criteria were established after machining. The slot profiles were first obtained using laser confocal microscopy; the original surface of the quartz wafer was defined as the reference line. The slot width was determined from the distance between the two intersection points formed by the left and right groove walls with the reference line. In addition, the slot depth was defined as the perpendicular distance between the deepest point of the slot and the reference line. A schematic illustration of the slot width and slot depth measurement is shown in Figure 7.

## 3. Results and Discussion

### 3.1. Effects of OCC and CPC on Quartz Wafer Cutting

**Figure 8:**
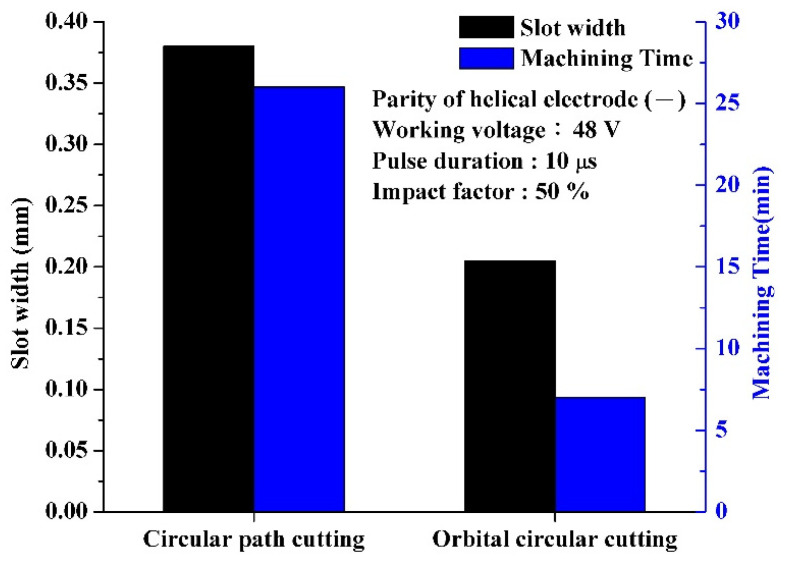
Effects of CPC and OCC on machining efficiencies and slot widths of quartz wafer.

**Figure 9:**
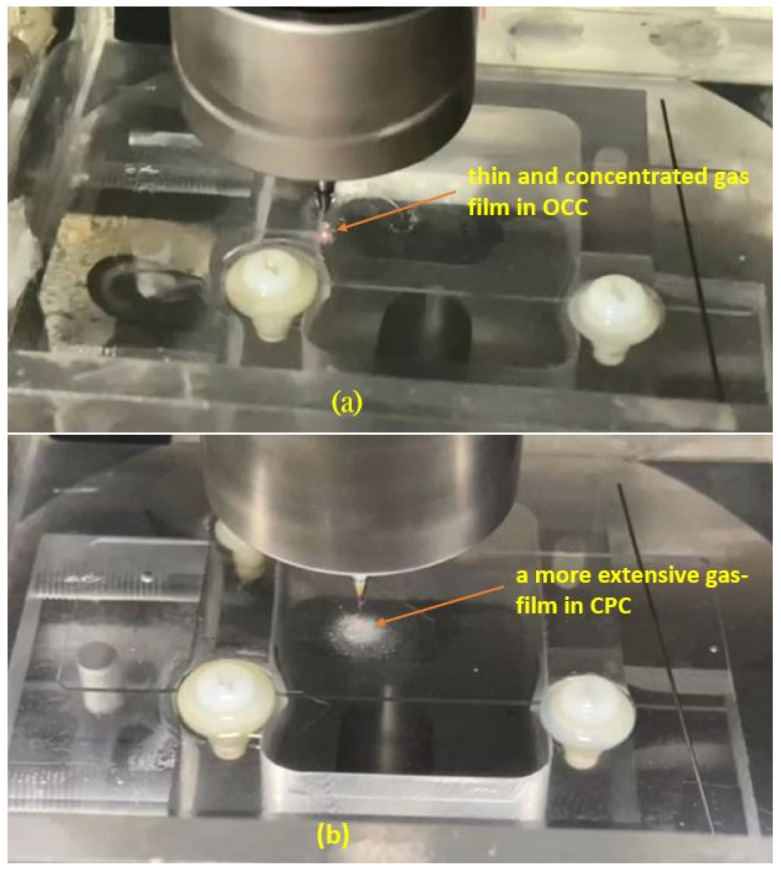
Photographs of cutting quartz wafer in (**a**) OCC and (**b**) CPC processes.

**Figure 10:**
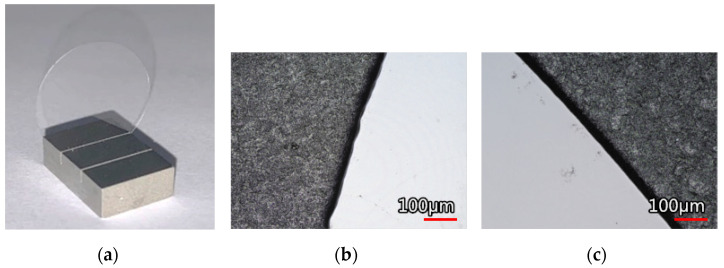
Circular quartz piece after cutting and morphological variations in cutting quartz edge during CPC and OCC: (**a**) circular quartz plate with 8 mm diameter; (**b**) cutting quartz edge by CPC; (**c**) cutting quartz edge using OCC.

To evaluate the effects of OCC and CPC on quartz wafer machining, a comparative study was conducted using ECDCM parameters listed in Table 1. For CPC, the process consisted of an initial drilling operation followed by circular path milling. In contrast, OCC employed continuous orbital cutting with a small incremental Z-axis feed. Therefore, the reported machining times represent the actual experimental durations from the start of machining until complete specimen separation. Figure 8 presents the machining performance of the two cutting strategies. The results show that the circular quartz plate could be completely separated within approximately 7 min using OCC. In contrast, CPC required about 26 min to complete the same cutting process. Therefore, the machining time of OCC was nearly four times shorter than that of CPC. As shown in Figure 9a, the OCC process removes material primarily using the drill tip of the helical electrode. The CCD image indicates that the gas film generated around the electrode tip is relatively thin and concentrated, suggesting a more stable discharge process and facilitating the continuous supply of fresh electrolyte into the machining zone. In contrast, Figure 9b shows that the CPC process removes material mainly through the cylindrical surface of the electrode. Because of the larger effective machining area, a more extensive gas film region is observed around the electrode, which suggests less stable discharge conditions and less efficient electrolyte renewal. Significant differences were also observed in slot width; the average slot width was 0.205 mm, corresponding to an overcut of approximately 55 μm when OCC was employed. In comparison, CPC produced a slot width of 0.380 mm, resulting in an overcut of approximately 230 μm. These results indicate that OCC substantially reduced material loss during the circular quartz piece cutting, yielding nearly a twofold improvement in material utilization compared with CPC. The material loss was evaluated based on the volume of material removed during cutting. Specifically, the removed volume was calculated as the difference between the outer and inner circular areas defined by the cutting slot, multiplied by the wafer thickness. The material loss reduction was then determined by comparing the removed volumes of the two processes. Figure 10 shows the fabricated 8 mm diameter circular quartz specimens and the corresponding edge morphologies obtained using the two cutting methods. As shown in Figure 10a, OCC successfully produced a complete circular quartz piece under the machining conditions listed in Table 1. The edge morphology of this specimen, fabricated by OCC, is presented in Figure 10b, whereas the machining edge shape obtained by CPC is shown in Figure 10c. It can be observed that the quartz cutting edge produced by OCC was significantly smoother than that obtained by CPC. This improvement can be attributed to the distinct material removal mechanisms of the two cutting strategies. Material removal was primarily performed by the electrode tip during OCC; consequently, the gas film generated on the electrode surface was more localized and stable, facilitating the concentration of the discharge energy within the machining zone. Furthermore, fresh electrolyte could be quickly supplied to the discharge region, promoting more stable machining conditions and improved cutting quality. In contrast, CPC relied mainly on the cylindrical surface of the electrode for material removal. The larger machining area increased the difficulty of maintaining a stable gas film, while electrolyte replenishment within the machining zone became less effective. As a result, discharge stability deteriorated, leading to a rougher edge shape and poorer cutting quality. Based on the superior machining efficiency, reduced overcut, and improved edge quality achieved by OCC, the following sections focus on the OCC to further investigate the effects of machining parameters and to establish an optimized method for high-quality quartz wafer cutting.

### 3.2. Effects of Working Voltage on Slot Geometry Cutting

**Figure 11:**
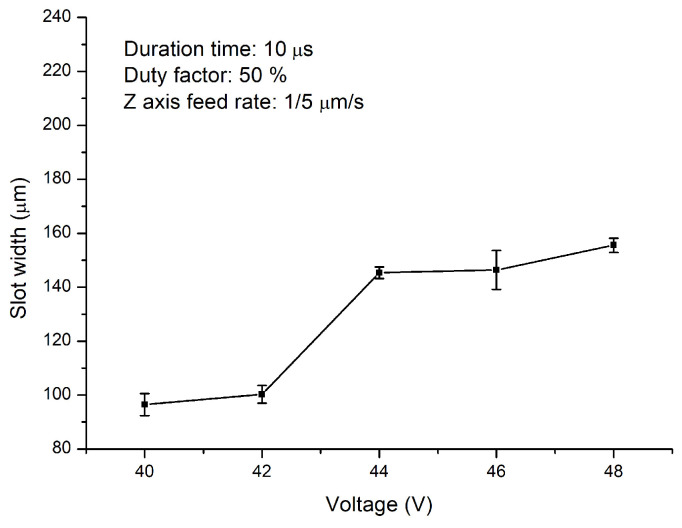
Effects of working voltage on cutting slot width.

**Figure 12:**
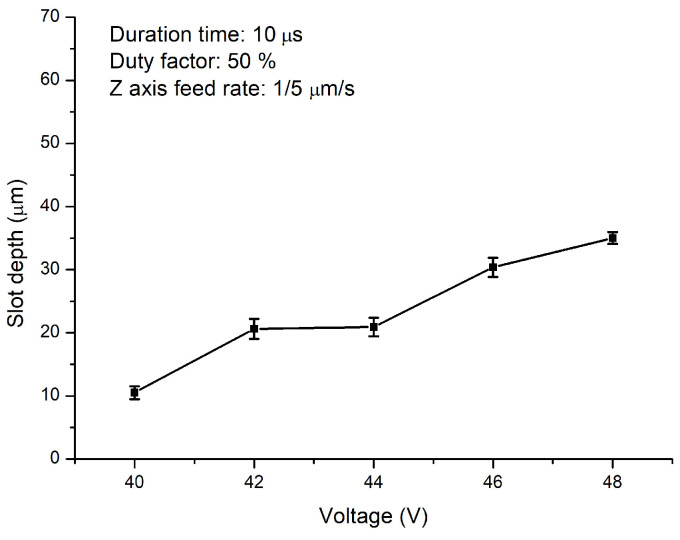
Effects of working voltage on cutting slot depth.

**Figure 13:**
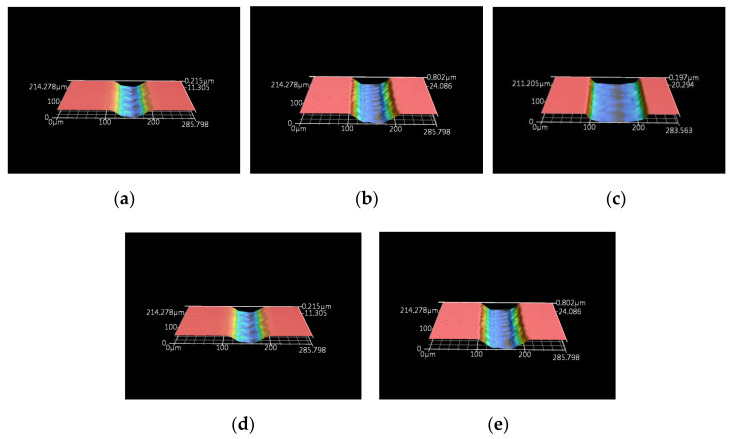
Cutting slot profile by OCC with voltages of (**a**) 40 V, (**b**) 42 V, (**c**) 44 V, (**d**) 46 V and (**e**) 48 V.

**Figure 14:**
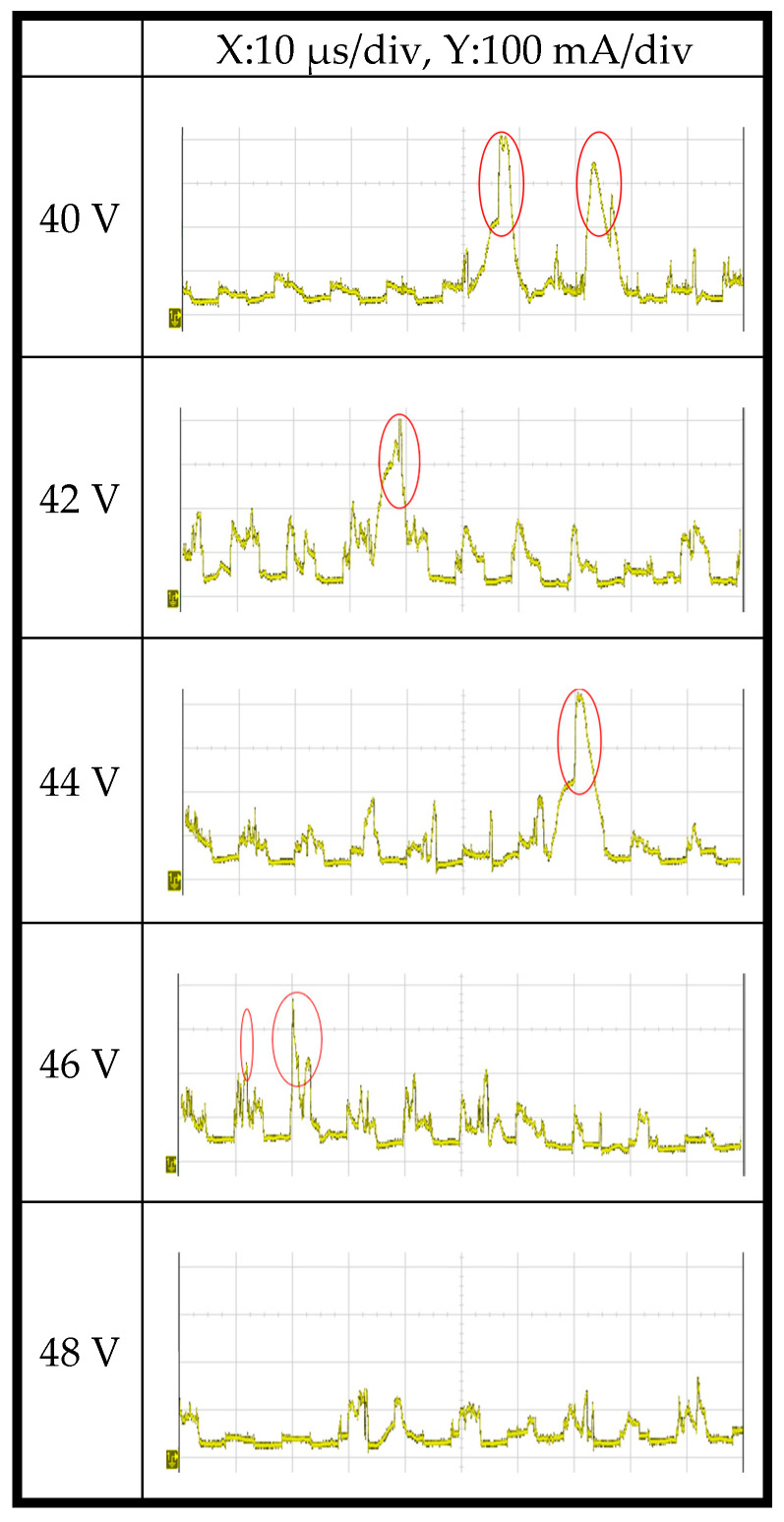
Current waves at different working voltages.

Since the applied voltage significantly influences gas film formation during the electrolysis process, the effect of working voltage on quartz wafer cutting performance was first investigated. During the experiments, the pulse period, Z-axis feed rate, and duty cycle were fixed at 10 μs, 1/5 μm/s, and 50%, respectively. Figure 11 shows the relationship between working voltage and slot width, while Figure 12 illustrates the effect of working voltage on slot depth. The results indicate that both slot width and slot depth increased with increasing working voltage. This behavior can be attributed to the enhanced gas generation rate at higher voltages. As the number of generated bubbles increased, a thicker and more stable insulating gas film was formed around the tool electrode. The stable gas film facilitated more frequent discharge events between the electrode and the quartz wafer, resulting in increased material removal and consequently larger slot dimensions. Figure 13 presents the slot profiles measured using the white-light interferometer under different working voltages. At working voltages ranging from 40 to 46 V, numerous dark regions can be observed on the slot bottom surface, as shown in Figure 13a–c. These features are attributed to pits generated by discharge erosion and chemical etching. When the applied voltage was relatively low, the resulting gas film exhibited a loose and unstable structure, leading to unstable discharge behavior and the formation of irregular pits on the machined surface. In contrast, when the working voltage was increased to 48 V, the slot bottom became noticeably smoother, as shown in Figure 13e. This result suggests that a denser and more stable gas film was established at 48 V, thereby improving discharge stability and enhancing machining quality. The stability of the insulating gas film was inferred from the measured machining current waveforms. Figure 14 shows the current waveforms obtained under different working voltages. At voltages between 40 and 46 V, the bubble generation rate was relatively low, causing bubbles to detach easily from the surface of the helical electrode before a stable gas film could be established. Consequently, direct contact between the electrode and the electrolyte frequently occurred, leading to electrolysis reactions. These electrolysis currents, indicated by the red markers in Figure 14, contributed to unstable material removal and are considered a major reason for the relatively rough slot surfaces observed at working voltages between 40 and 46 V. In contrast, at a working voltage of 48 V, the machining current remained stable throughout the process, and no noticeable electrolysis current was observed in Figure 14. This indicates that a stable insulating gas film was established, allowing the discharge energy to be more effectively concentrated within the machining zone. Consequently, a higher effective current density and discharge efficiency were achieved, leading to increased material removal and larger groove width and depth.

### 3.3. Effects of Pulse Duration on Slot Geometry Cutting

**Figure 15:**
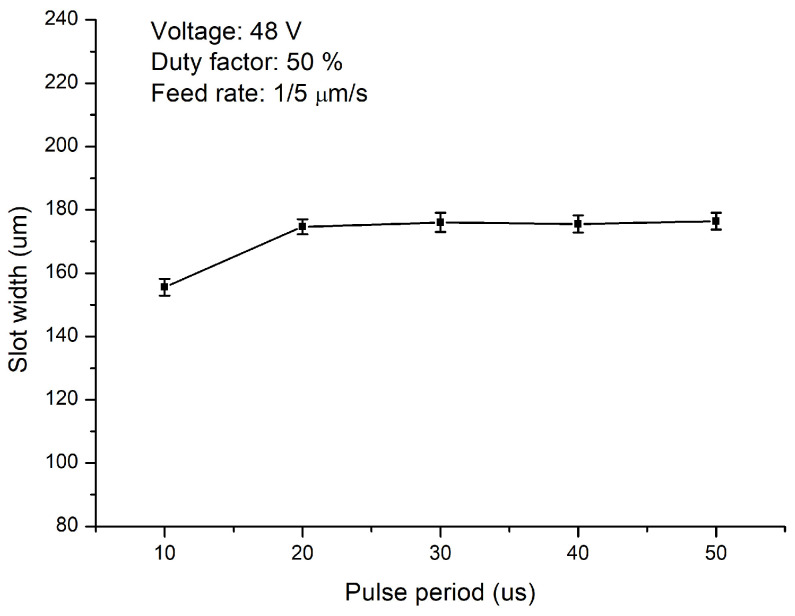
Effects of pulse duration on cutting slot width.

**Figure 16:**
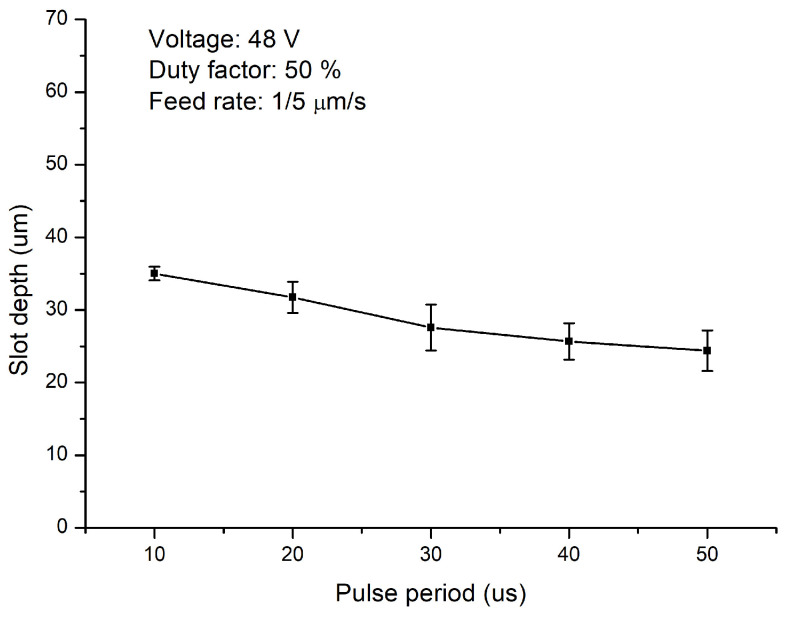
Effects of pulse duration on cutting slot depth.

**Figure 17:**
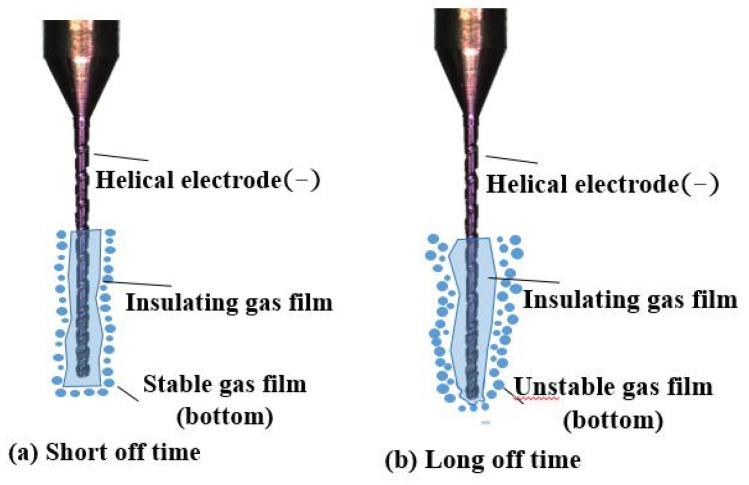
Changeable gas film at different off times.

**Figure 18:**
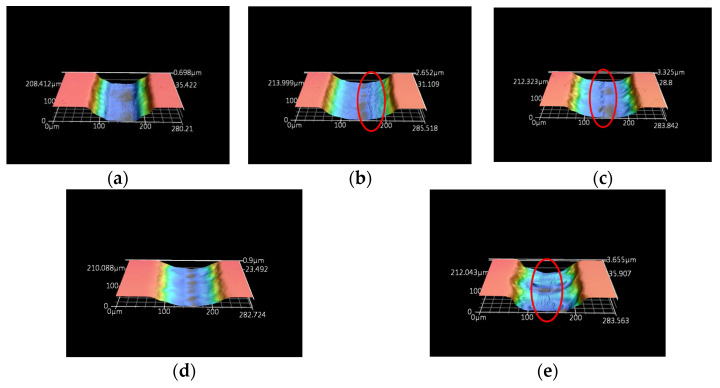
Cutting slot profile using OCC with pulse durations of (**a**) 10 µs, (**b**) 20 µs, (**c**) 30 µs, (**d**) 40 µs and (**e**) 50 µs.

Pulse duration determines the discharge time during quartz wafer cutting and therefore plays an important role in the formation of slot geometry. To investigate its effect, the working voltage, Z-axis feed rate, and duty cycle were fixed at 48 V, 0.2 μm/s (1/5 μm/s), and 50%, respectively. Figure 15 shows the relationship between pulse duration and slot width, while Figure 16 presents the effect of pulse duration on slot depth. As shown in Figure 15, the slot width increased noticeably when the pulse duration was increased from 10 μs to 20 μs. However, further increases in pulse duration resulted in only a slight increase in slot width. This behavior can be attributed to the short discharge duration of 10 μs, limiting the discharge energy and consequently reducing the overcut. Since the duty factor was maintained at 50%, increasing the pulse duration simultaneously increased the pulse off time. Under these conditions, the gas film surrounding the helical electrode had more time to expand outward. As a result, a thicker gas film formed along the electrode sidewall before the arrival of the next pulse, facilitating sidewall discharges and increasing the slot width. Nevertheless, as the off time continued to increase, bubble transport became more pronounced, leading to faster gas movement around the electrode. Consequently, the discharge intensity could not increase effectively with pulse duration, resulting in only a small increase in slot width. The results shown in Figure 16 indicate that slot depth decreased slightly with increasing pulse duration. This phenomenon is also associated with the increase in pulse off time. The thicker gas film generated along the electrode sidewall promoted stronger turbulent bubble motion, as illustrated in Figure 17. Such turbulence interfered with the formation of a dense and stable gas film at the electrode tip, thereby reducing the efficiency of downward discharge machining and limiting material removal in the depth direction. Figure 18 presents the slot profiles measured under different pulse durations. Pronounced etching marks were observed at the slot bottoms when pulse durations of 20, 30, and 50 μs were employed, as highlighted by the red circles in the figure. These surface features are attributed to instability in the gas film formed at the electrode tip. As the off time increased, fluctuations in gas film thickness became more severe, causing variations in discharge intensity and leaving irregular discharge etching traces on the slot bottom. In contrast, when a pulse duration of 10 μs was used, the shorter off time promoted the formation of a more uniform gas film. Consequently, the slot bottom exhibited a smoother and flatter morphology, with no obvious discharge etching marks observed after machining.

### 3.4. Effects of Z-Axis Feed Rate on Slot Geometry Cutting

**Figure 19:**
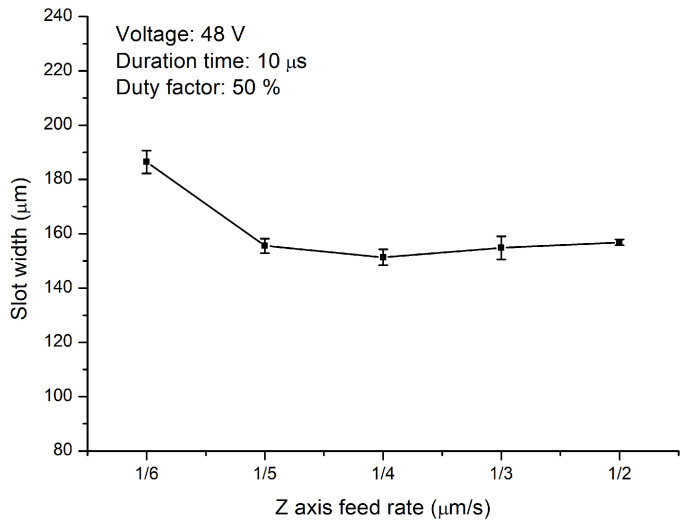
Effects of Z-axis feed rate on cutting slot width.

**Figure 20:**
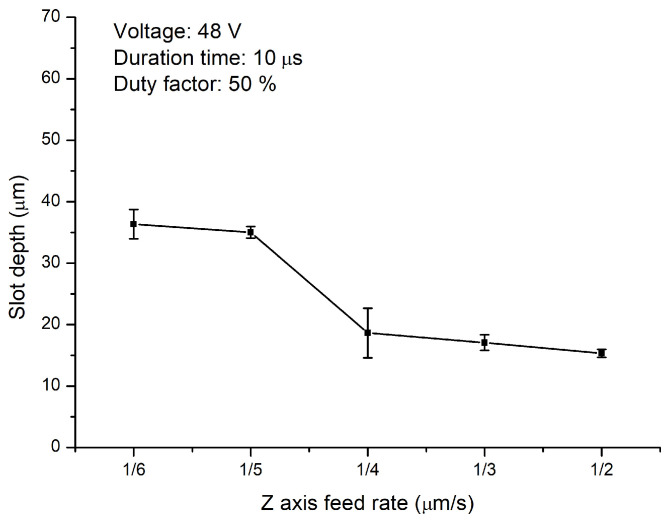
Effects of Z-axis feed rate on cutting slot depth.

**Figure 21:**
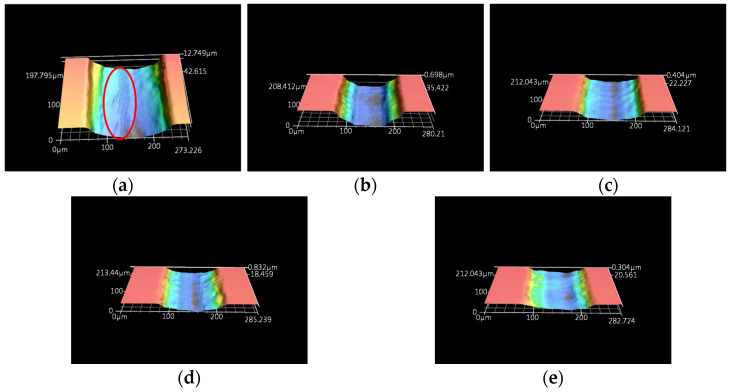
Cutting slot profile using OCC with Z-axis feed rates of (**a**) 1/6 µm/s, (**b**) 1/5 µm/s, (**c**) 1/4 µm/s, (**d**) 1/3 µm/s and (**e**) 1/2 µm/s.

**Figure 22:**
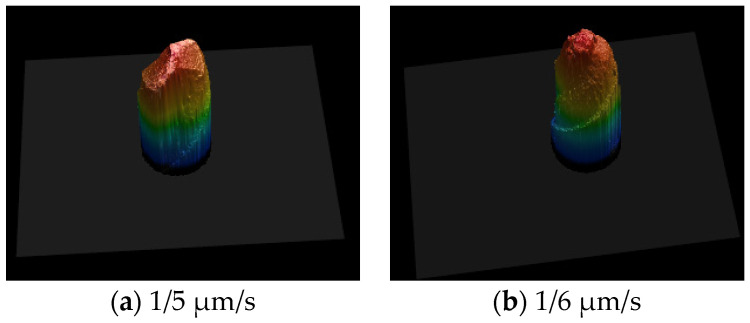
Electrode tip shape after OCC at different Z-axis feed rates.

The Z-axis feed rate has a significant influence on the efficiency of the machined slots; therefore, the effects of Z-axis feed rate on slot width and slot depth during quartz wafer cutting were investigated. In these experiments, the working voltage, pulse duration, and duty cycle were fixed at 48 V, 10 μs, and 50%, respectively. Figure 19 shows the effect of Z-axis feed rate on slot width, while Figure 20 illustrates the relationship between Z-axis feed rate and slot depth. The results indicate that both slot width and slot depth decreased with increasing Z-axis feed rate. The reason can be attributed to a thicker and denser insulating gas film being formed around the electrode when the electrode advances more slowly, resulting in more stable discharge conditions and higher discharge energy. Consequently, greater material removal occurred, leading to increased slot width and slot depth. Conversely, increasing the feed rate reduced the duration of discharge action at a given location, thereby limiting material removal and reducing slot dimensions. Figure 21 presents the slot morphologies obtained under different Z-axis feed rates. The 3D profiles reveal that no obvious etching traces were observed on the slot bottom when the feed rate decreased from 1/2 μm/s to 1/5 μm/s. However, when the feed rate was further reduced to 1/6 μm/s, the machining process became considerably slower, promoting the formation of a thicker and denser insulating gas film around the electrode. Under these conditions, discharge activity became more intense, and the larger vaporization and explosion pressures generated during discharge resulted in noticeable etching marks on the slot surface, as highlighted by the red circle in Figure 19. In addition to affecting slot morphology, the Z-axis feed rate also influenced electrode wear. Figure 22 shows the three-dimensional profiles of spiral tool electrodes after machining under different feed rate conditions. As shown in Figure 22a, when a feed rate of 1/5 μm/s was employed, the electrode tip retained its original drill bit geometry after quartz wafer cutting. In contrast, when the feed rate was reduced to 1/6 μm/s, severe electrode wear was observed, and the electrode tip gradually evolved into a pointed shape, as shown in Figure 22b. These results indicate that although a lower feed rate can facilitate complete cutting of the quartz wafer, excessive discharge energy and prolonged machining duration lead to overcutting of the slot and accelerate the electrode wear. Consequently, the dimensional accuracy and cutting precision of the machining process are adversely affected.

### 3.5. Effects of Duty Factor on Slot Geometry Cutting

**Figure 23:**
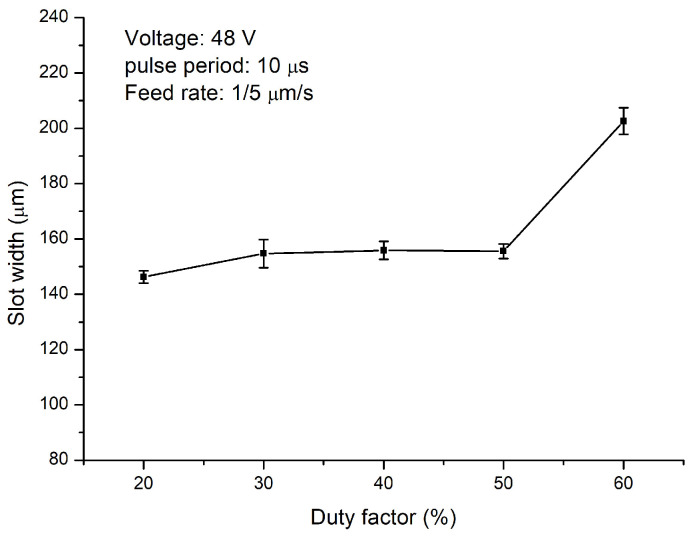
Effects of duty factor on cutting slot width.

**Figure 24:**
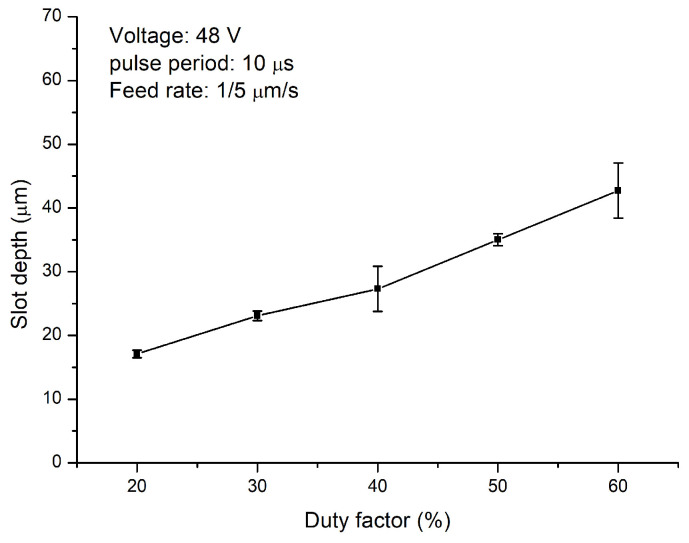
Effects of duty factor on cutting slot depth.

**Figure 25:**
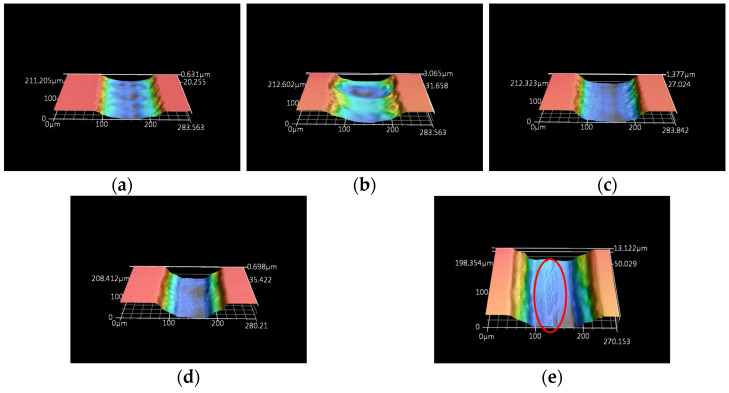
Cutting slot profile by OCC with duty factors of (**a**) 20%, (**b**) 30%, (**c**) 40%, (**d**) 50% and (**e**) 60%.

Since the duty factor directly influences the discharge energy during ECDCM, it plays an important role in determining the cutting accuracy of quartz wafers. To investigate its effect, the working voltage, pulse duration, and Z-axis feed rate were fixed at 48 V, 10 μs, and 1/5 μm/s, respectively. Figure 23 shows the effect of duty factor on slot width, while Figure 24 presents the relationship between duty factor and slot depth. The results indicate that both slot width and slot depth increased with increasing duty factor. This trend can be attributed to the increase in discharge energy associated with a higher duty factor. As the discharge energy increased, the material removal of OCC was enhanced, resulting in greater slot dimensions after machining. Figure 25 presents the slot profiles obtained under different duty factor conditions. From 3D surface profiles, numerous dark regions can be observed on the slot bottoms when duty factors of 20% and 30% were employed, as shown in Figure 25a,b. These features correspond to pits formed by the combined effects of discharge heating and chemical etching. When the duty cycle was relatively low, the pulse off time became excessively long, leading to the formation of a loose and unstable insulating gas film, as shown in Figure 15. The unstable gas film reduced discharge stability and resulted in incomplete material removal, thereby producing irregular pits on the slot bottom. As the duty cycle increased from 40% to 50%, the formation of the insulating gas film became more stable, facilitating a more consistent discharge process. Consequently, the slot bottoms exhibited smoother and flatter morphologies after machining, as shown in Figure 25c,d. These results suggest that an appropriate duty factor can improve discharge stability and enhance machining quality. However, when the duty factor was further increased to 60%, the discharge energy became excessively high. Under these conditions, intense thermal loading was generated during the OCC process, resulting in the formation of distinct discharge-induced cracks on the groove bottom, as highlighted by the red circle in Figure 25e. Therefore, although increasing the duty factor enhanced material removal and increased slot dimensions, an excessively high duty factor adversely affected surface integrity and deteriorated the machining quality of the quartz wafer.

### 3.6. Validation of Optimized Machining Parameters for Circular Quartz Specimen Cutting

**Figure 26:**
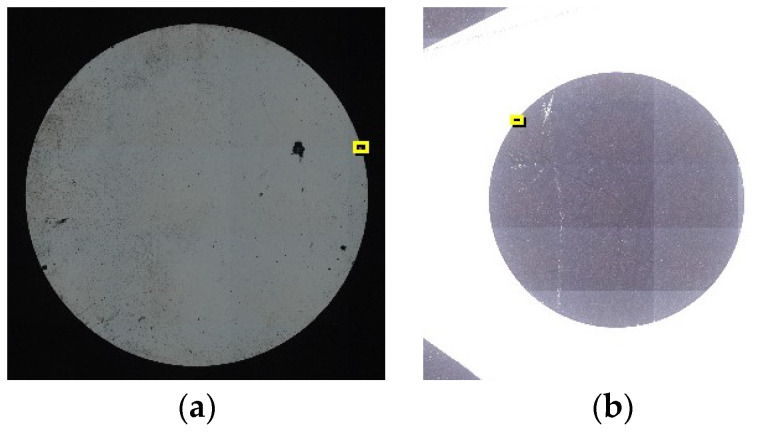
Circular cutting profile of quartz plate with 8 mm diameter. (**a**) Top view of circular quartz piece. (**b**) Top view of cutting hole in quartz wafer.

Following the investigation of the effects of working voltage, pulse duration, Z-axis feed rate, and duty factor on quartz wafer cutting, the optimal machining parameters were applied to evaluate their capability for fabricating circular quartz specimens. During the validation experiments, the Z-axis was programmed to cut to a depth of 90 μm, and the machining process was automatically terminated once complete separation of the circular quartz specimen was achieved. The results demonstrated that a working voltage of 48 V, a pulse duration of 10 μs, a Z-axis feed rate of 1/5 μm/s and a duty factor of 50% produced the most favorable cutting performance. Under these machining conditions, a circular quartz specimen with a smooth edge morphology and an outer diameter of 8.069 mm was successfully fabricated, as shown in Figure 26. For the working voltage, when lower voltages of 44 V and 46 V were employed, the insulating gas film generated around the electrode was insufficiently dense and stable to sustain continuous discharge activity. Consequently, only partial penetration of the circular cutting path was achieved. In some cases, the partially separated quartz specimen was forced downward during machining, resulting in electrode fracture before complete cutting could be accomplished. When a pulse duration of 20 μs was used, the corresponding increase in pulse off time adversely affected the stability of gas film formation. As a result, discharge efficiency decreased, and only partial penetration of the circular cutting path was obtained. The unstable machining condition eventually led to electrode breakage before the quartz specimen could be completely separated. Although a lower Z-axis feed rate of 1/6 μm/s enabled complete separation of the circular quartz specimen, the prolonged machining time increased the overcut and made dimensional control more difficult. Furthermore, the excessive material removal reduced the overall machining accuracy of the cutting process. When a duty factor of 60% was applied, the discharge energy increased substantially, leading to accelerated electrode wear. The resulting deformation of the electrode altered the gas film formation behavior and prevented the establishment of a stable insulating gas layer around the electrode surface. Consequently, discharge stability deteriorated, and only partial penetration of the circular cutting path was achieved. Continued machining under these conditions ultimately resulted in electrode fracture. Based on the above results, the combination of a 48 V working voltage, 10 μs pulse duration, 1/5 μm/s Z-axis feed rate, and 50% duty factor was identified as the optimal parameter set for quartz wafer cutting. These conditions provided stable discharge behavior, minimized electrode wear, and enabled the successful fabrication of circular quartz specimens with high dimensional accuracy and good edge quality.

## 4. Conclusions

In this study, a micro-helical electrode made of tungsten carbide was employed to perform orbital ECDCM for quartz wafer cutting. The experimental results demonstrated that OCC using a helical electrode is a feasible and efficient method for fabricating circular quartz specimens. The major findings are summarized as follows:

1. Compared with CPC, OCC significantly improved the machining efficiency; the time required to fabricate an 8 mm diameter circular quartz specimen was reduced by approximately four times, while material loss was reduced by nearly twofold.
2. During the OCC of quartz wafers, increasing the working voltage promoted the formation of a thicker and denser insulating gas film around the electrode, resulting in higher discharge energy. Consequently, both slot width and slot depth increased with increasing working voltage.
3. Increasing the pulse duration led to an increase in slot width. However, the longer pulse off time associated with a larger pulse duration promoted the lateral diffusion of the insulating gas film from the electrode tip toward the sidewall, reducing the stability of gas film formation at the electrode tip. As a result, the downward discharge efficiency decreased, leading to a reduction in slot depth.
4. Both slot width and slot depth decreased with increasing Z-axis feed rate. A higher feed rate reduced the formation time of the insulating gas film and limited discharge energy generation. However, when the feed rate was reduced to 1/6 μm/s, the prolonged gas film formation intensified discharge energy, resulting in noticeable etching marks on the slot bottom.
5. Increasing the duty factor enhanced the discharge energy during ECDM, thereby increasing both slot width and slot depth. When the duty factor reached 50%, a stable insulating gas film was established, producing a smoother and flatter slot bottom morphology.
6. The optimal machining parameters for quartz wafer cutting were identified as a working voltage of 48 V, a pulse duration of 10 μs, a Z-axis feed rate of 1/5 μm/s, and a duty factor of 50%. Under these conditions, a circular quartz specimen with a diameter of 8.069 mm was successfully fabricated with good dimensional accuracy and edge quality.

Since in-process electrical monitoring can improve machining stability and machining efficiency [19]. The future research of this study will focus on developing an ECDM system with servo-controlled electrode feeding and integrating real-time process monitoring techniques, including electrical sensing and other advanced sensing methods, to evaluate machining stability, optimize the discharge process, and further improve machining performance.

## Figures and Tables

**Table 1:** Cutting parameters for circular quartz piece during OCC and CPC.

Experimental Parameters	Present Parameters
parity of helical electrode	(—)
Helical electrode size (µm)	150
Working voltage (V)	48
Pulse duration (µs)	10
duty factor (%)	50
electrolyte concentration and volumetric ratio (M)	5M KOH:C_2_H_5_OH = 9:1

**Table 2:** Experimental parameters of OCC.

Experimental Parameters	Present Parameters
Working voltages (V)	40, 42, 44, 46, 48
Pulse durations (µs)	10, 20, 30, 40, 50
Feed rates of Z-axis (µm/s)	1/2, 1/3, 1/4, 1/5, 1/6
duty factors (%)	20, 30, 40, 50, 60

## Data Availability

The original contributions presented in this study are included in the article. Further inquiries can be directed to the corresponding author.
